# Supplementary material for: Low-Symmetry Phthalocyanines Bearing Carboxy-Groups: Synthesis, Spectroscopic and Quantum-Chemical Characterization
Source: Molecules. 2022 Jan 14;27(2):524. doi: 10.3390/molecules27020524 (PMC8781019; doi:10.3390/molecules27020524)
Supplement: Supplementary file 1 [file molecules-27-00524-s001.zip › molecules-1530628-supplementary.pdf]

Supporting information

Low-symmetry phthalocyanines bearing carboxy-groups: synthesis, spectroscopic and quantum-chemical characterization

Dmitry A. Bunin[a], Nobuhle Ndebele[b], Alexander G. Martynov[a], John Mack[b], Yulia G. Gorbunova[a,c], Tebello Nyokong[b]

[a] A. N. Frumkin Institute of Physical Chemistry and Electrochemistry, Russian Academy of Sciences, Leninsky pr., 31, building 4, Moscow 119071, Russia

[b] Institute for Nanotechnology Innovation, Department of Chemistry, Rhodes University, Makhanda 6140, South Africa

[c] N. S. Kurnakov Institute of General and Inorganic Chemistry, Russian Academy of Sciences, Leninsky pr., 31, Moscow 119991, Russia

Table of contents

^{1}H-NMR spectra 3

Figure S1. ^{1}H-NMR spectrum of **ZnPc1** 3

Figure S2. ^{1}H-NMR spectrum of **ZnPc2** 4

Figure S3. ^{1}H-NMR spectrum of **ZnPc3** 5

Figure S4. ^{1}H-NMR spectrum of **ZnPc4** 6

Figure S5. ^{1}H-NMR spectrum of **ZnPc*** 7

MALDI TOF mass-spectra 8

Figure S6. MALDI TOF mass-spectrum of **ZnPc1** 8

Figure S7. MALDI TOF mass-spectrum of **ZnPc2** 9

Figure S8. MALDI TOF mass-spectrum of **ZnPc3** 10

Figure S9. MALDI TOF mass-spectrum of **ZnPc4** 11

Figure S10. MALDI TOF mass-spectrum of **ZnPc*** 12

Concentration dependence of UV-Vis 13

Figure S11. UV-Vis spectra at the various concentration of **ZnPc1** in DMF 13

Figure S12. UV-Vis spectra at the various concentration of **ZnPc2** in DMF 14

Figure S13. UV-Vis spectra at the various concentration of **ZnPc3** in DMF 15

Figure S14. UV-Vis spectra at the various concentration of **ZnPc4** in DMF 16

Figure S15. UV-Vis spectra at the various concentration of **ZnPc*** in DMF 17

Cartesian coordinates of computed structures 18

Table S1. Gas-phase geometry of **ZnPc1** optimized at B3LYP/6-31G(d) level of theory 18

Table S2. Gas-phase geometry of **ZnPc2** optimized at B3LYP/6-31G(d) level of theory 23

Table S3. Gas-phase geometry of **ZnPc3** optimized at B3LYP/6-31G(d) level of theory 27

Table S4. Gas-phase geometry of **ZnPc4** optimized at B3LYP/6-31G(d) level of theory 30

Table S5. Gas-phase geometry of **ZnPc*** optimized at B3LYP/6-31G(d) level of theory 33

^{1}H-NMR spectra

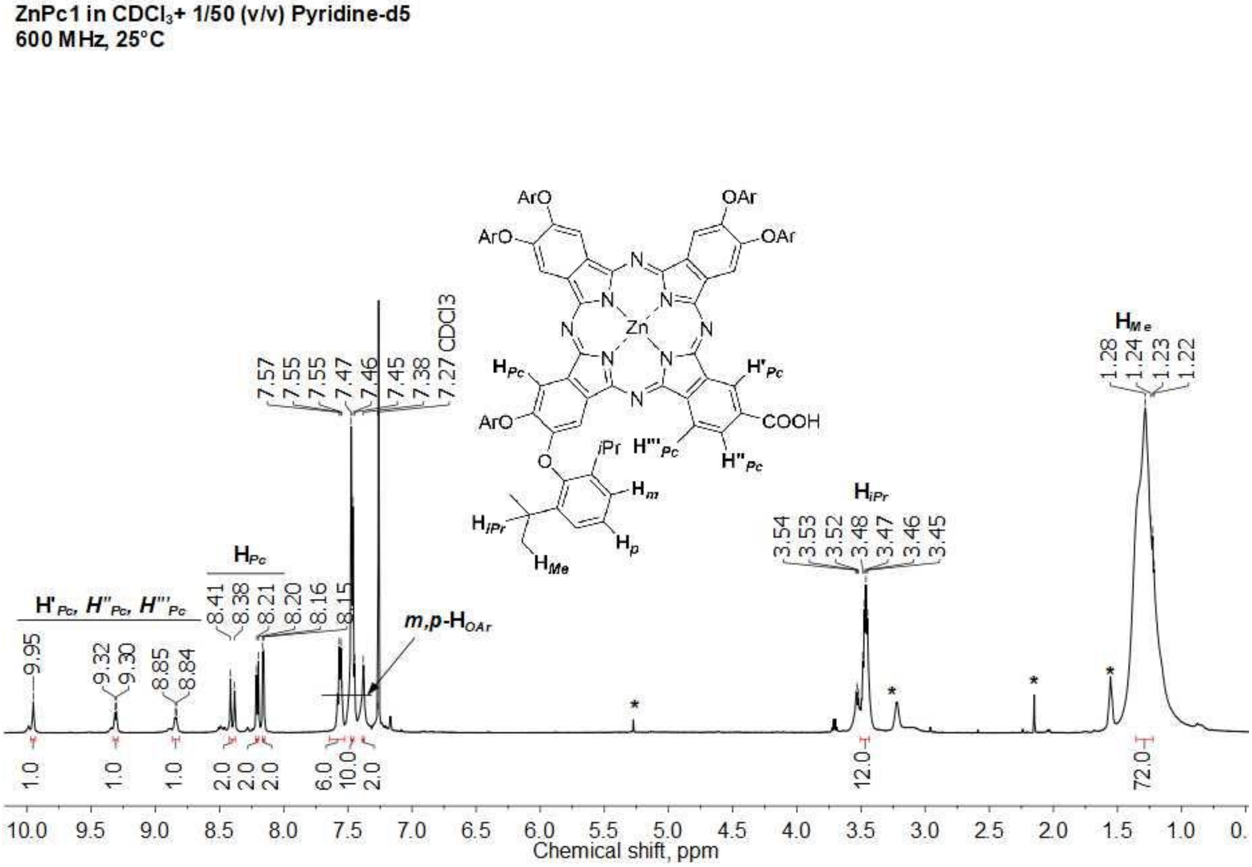

Figure S1. ^{1}H-NMR spectrum of **ZnPc1**
The asterisks indicate impurity signals

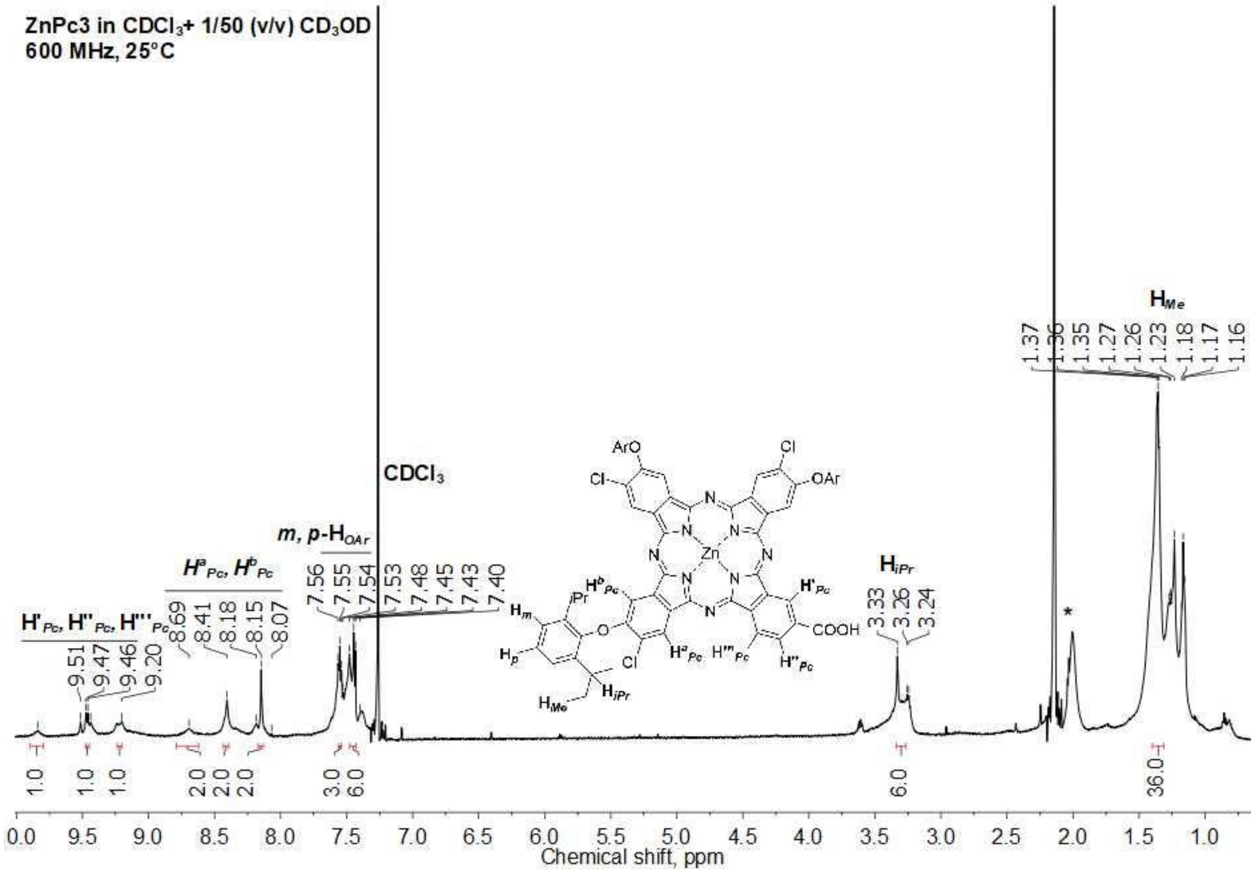

Figure S2. ^{1}H-NMR spectrum of **ZnPc2**

The asterisks indicate impurity signals

ZnPc2 in $CDCl_3$+ 1/50 (v/v) Pyridine-d5
300 MHz, 25°C

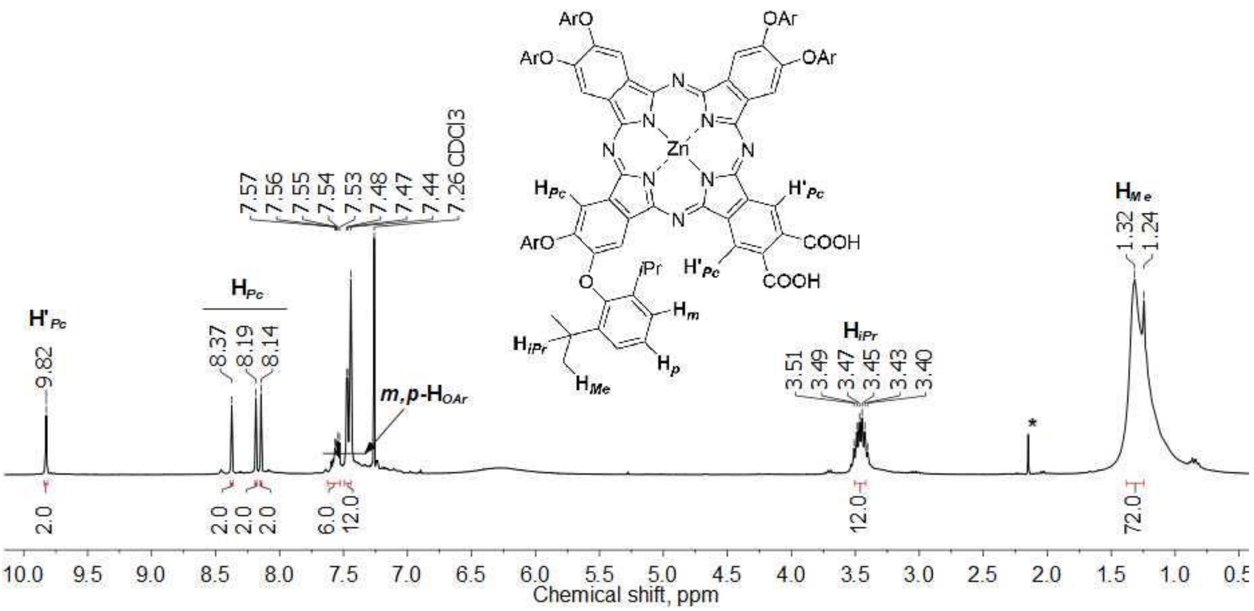

Figure S3. ^{1}H-NMR spectrum of **ZnPc3**
The asterisks indicate impurity signals

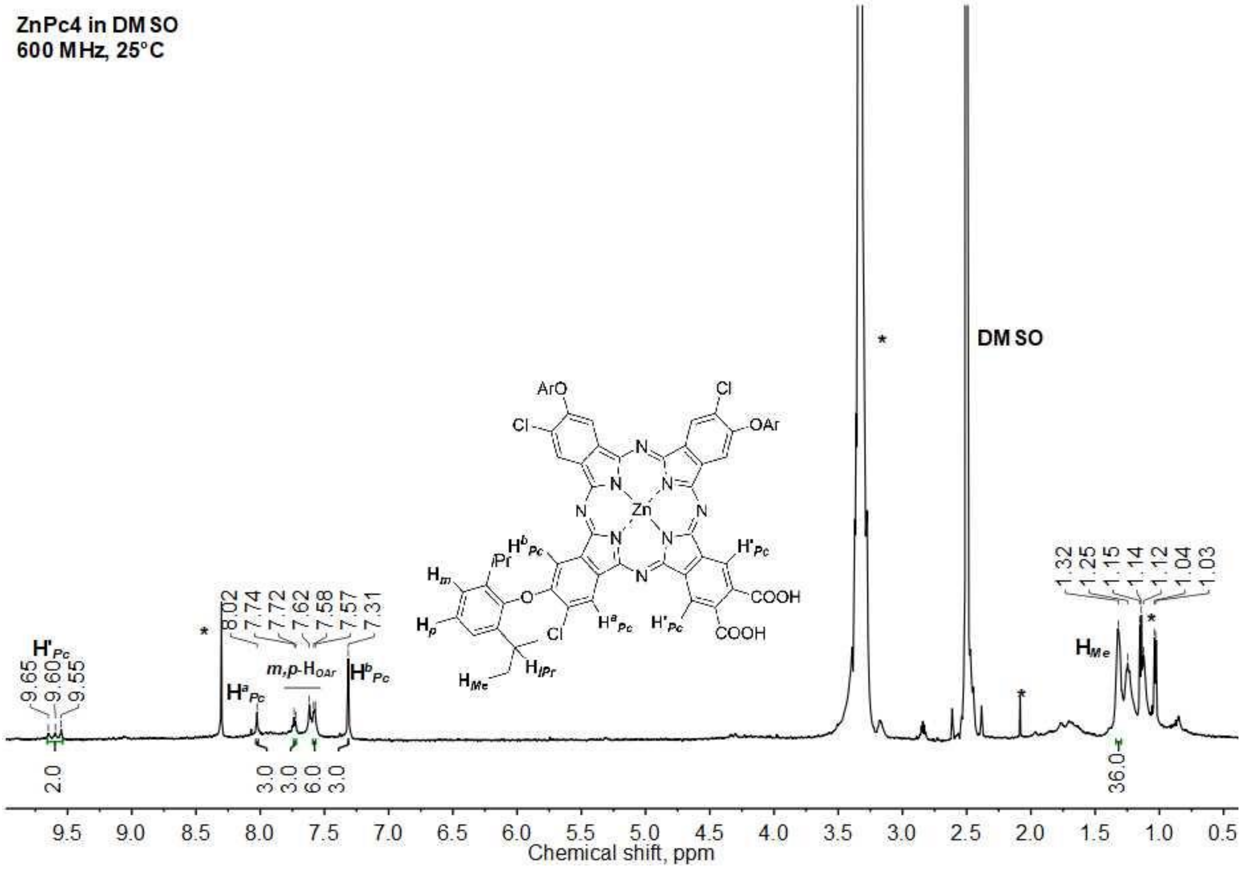

Figure S4. ^{1}H-NMR spectrum of **ZnPc4**

The asterisks indicate impurity signals

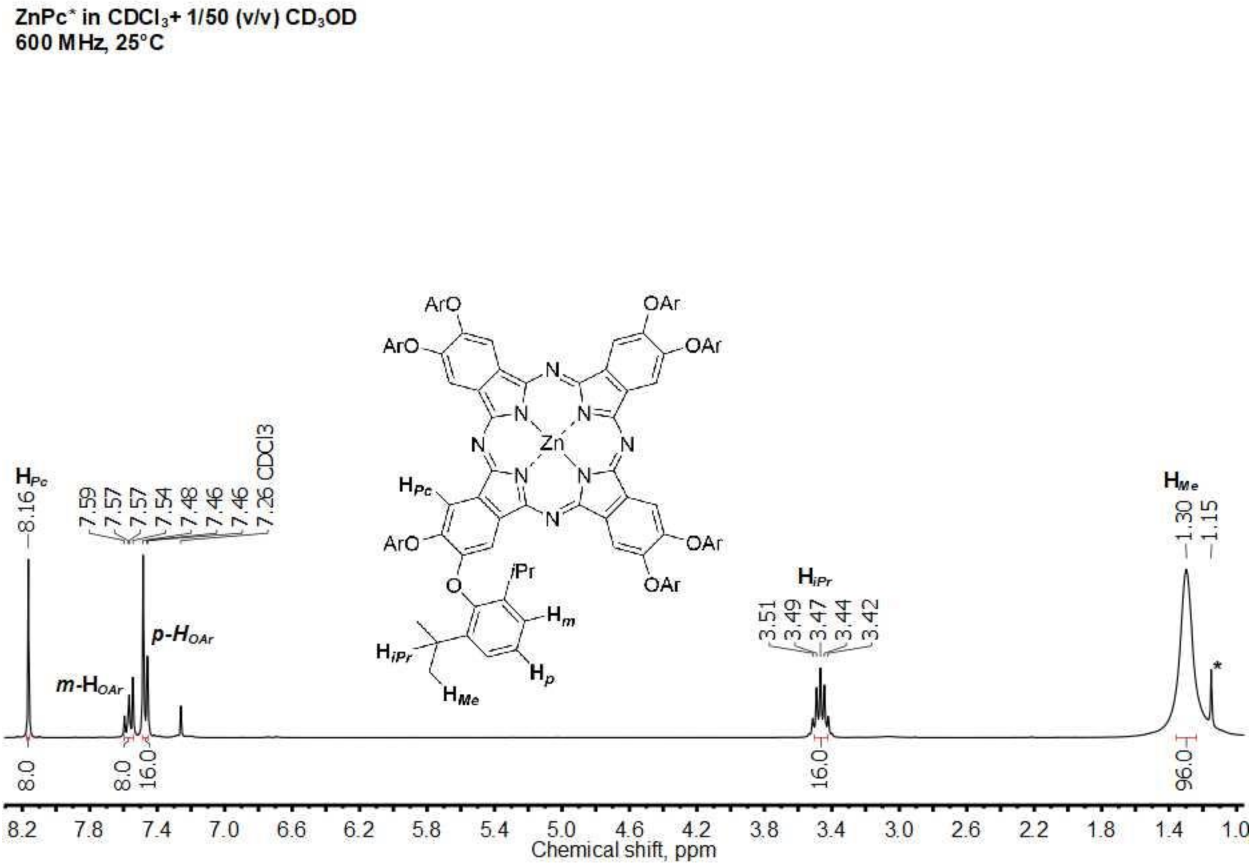

Figure S5. ^{1}H-NMR spectrum of **ZnPc***

The asterisks indicate impurity signals

MALDI TOF mass-spectra

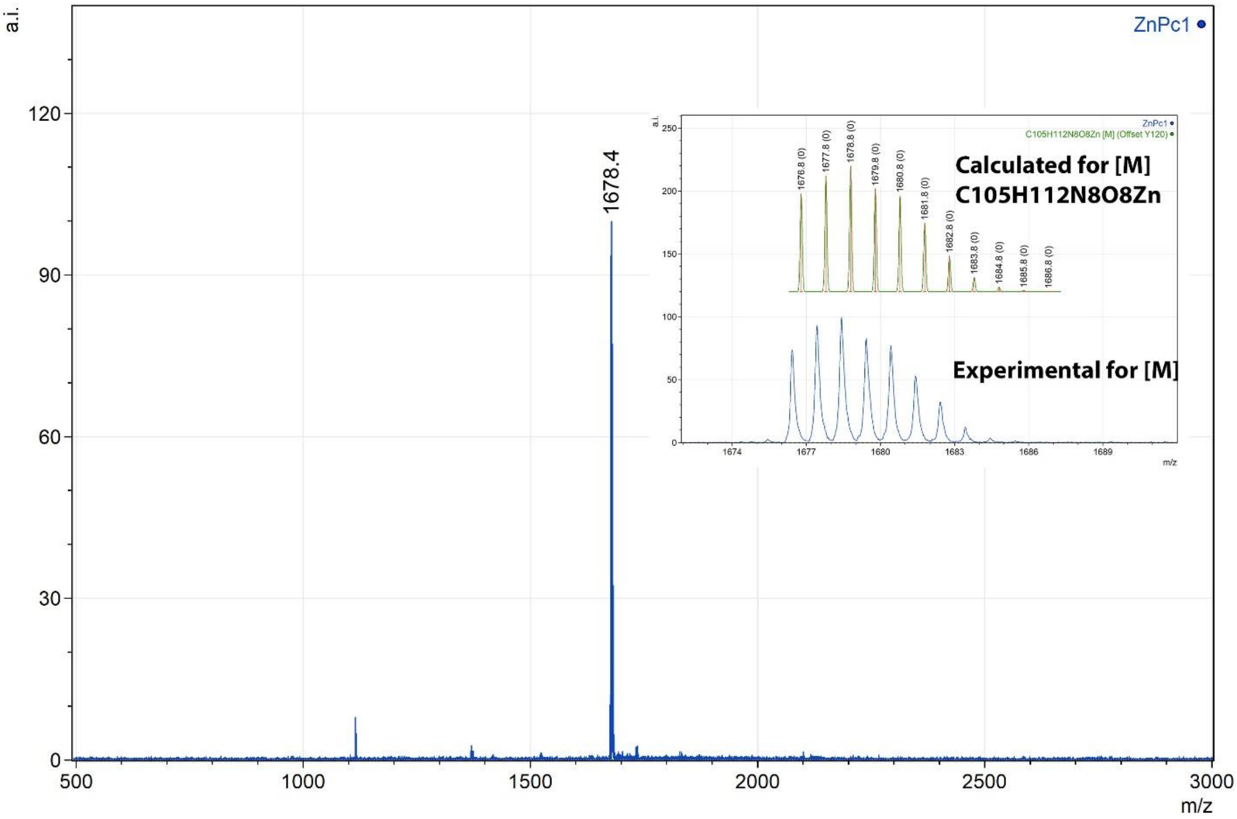

Figure S6. MALDI TOF mass-spectrum of **ZnPc1**

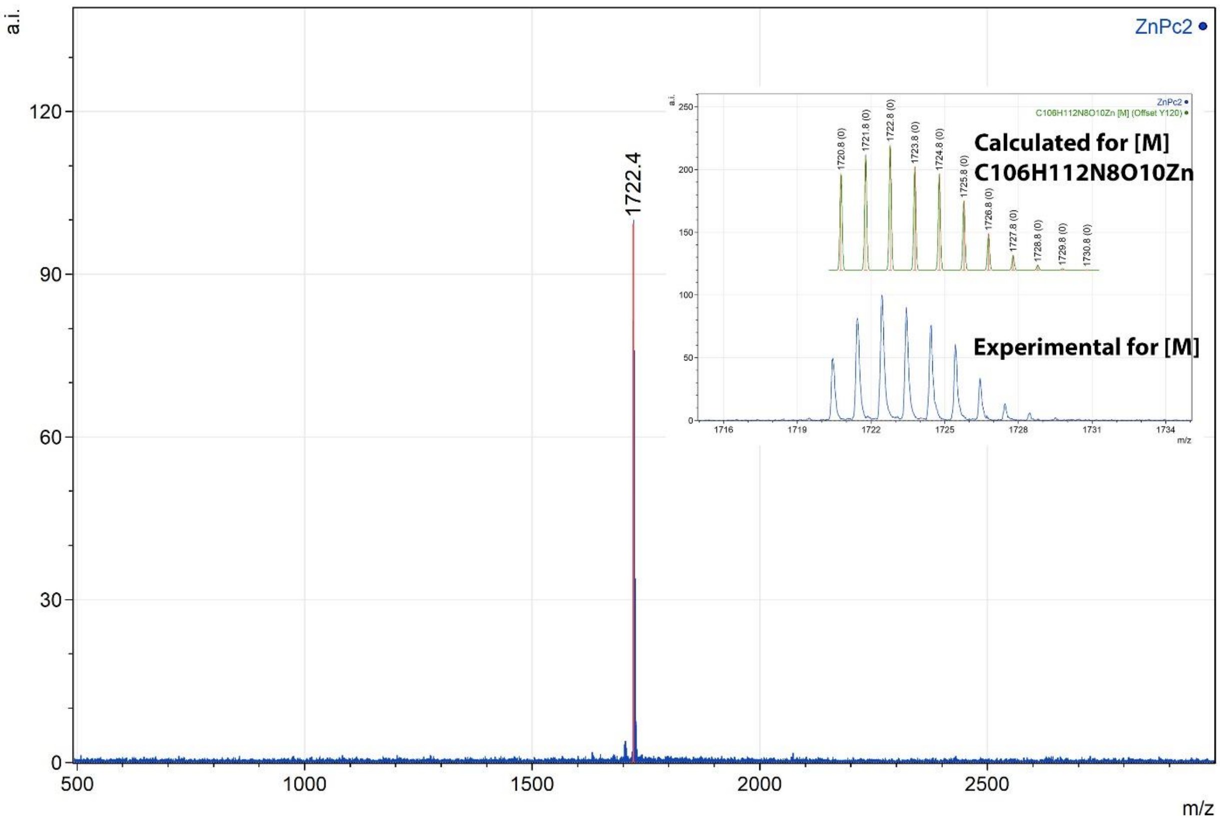

Figure S7. MALDI TOF mass-spectrum of **ZnPc2**

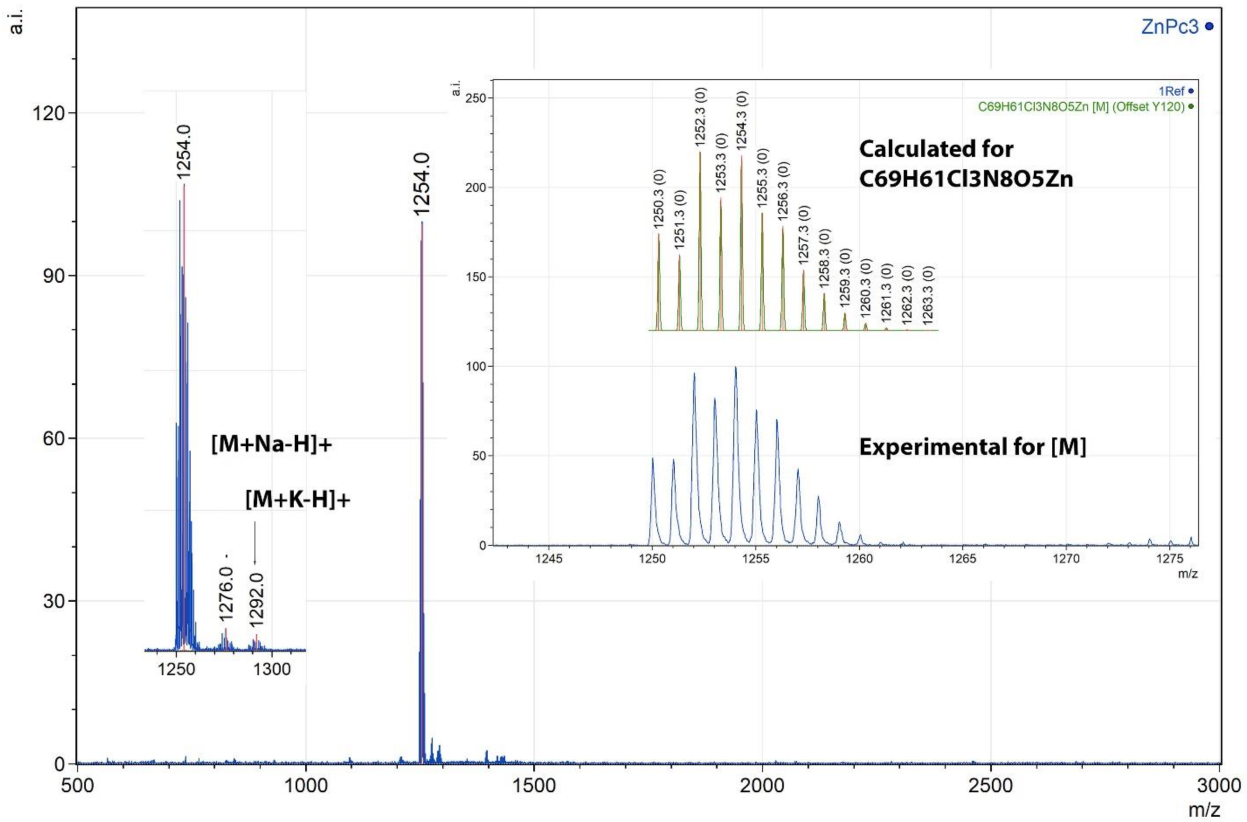

Figure S8. MALDI TOF mass-spectrum of **ZnPc3**

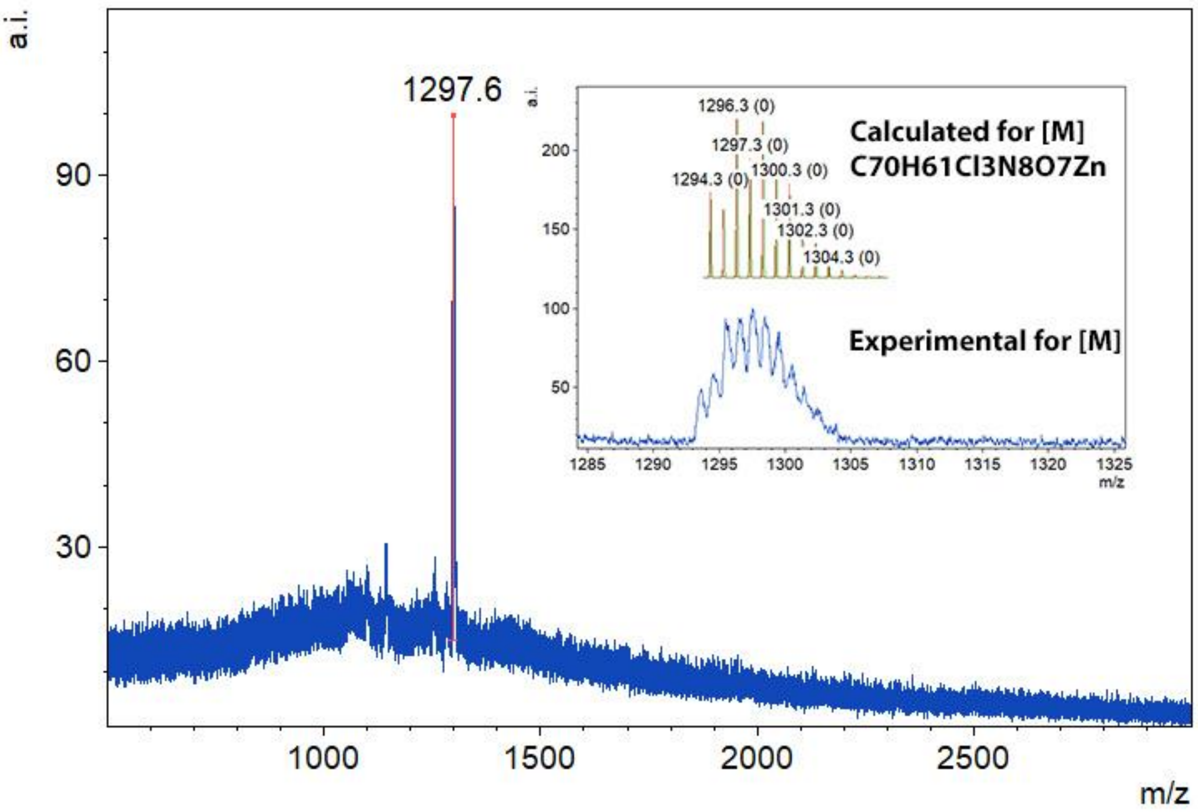

Figure S9. MALDI TOF mass-spectrum of **ZnPc4**

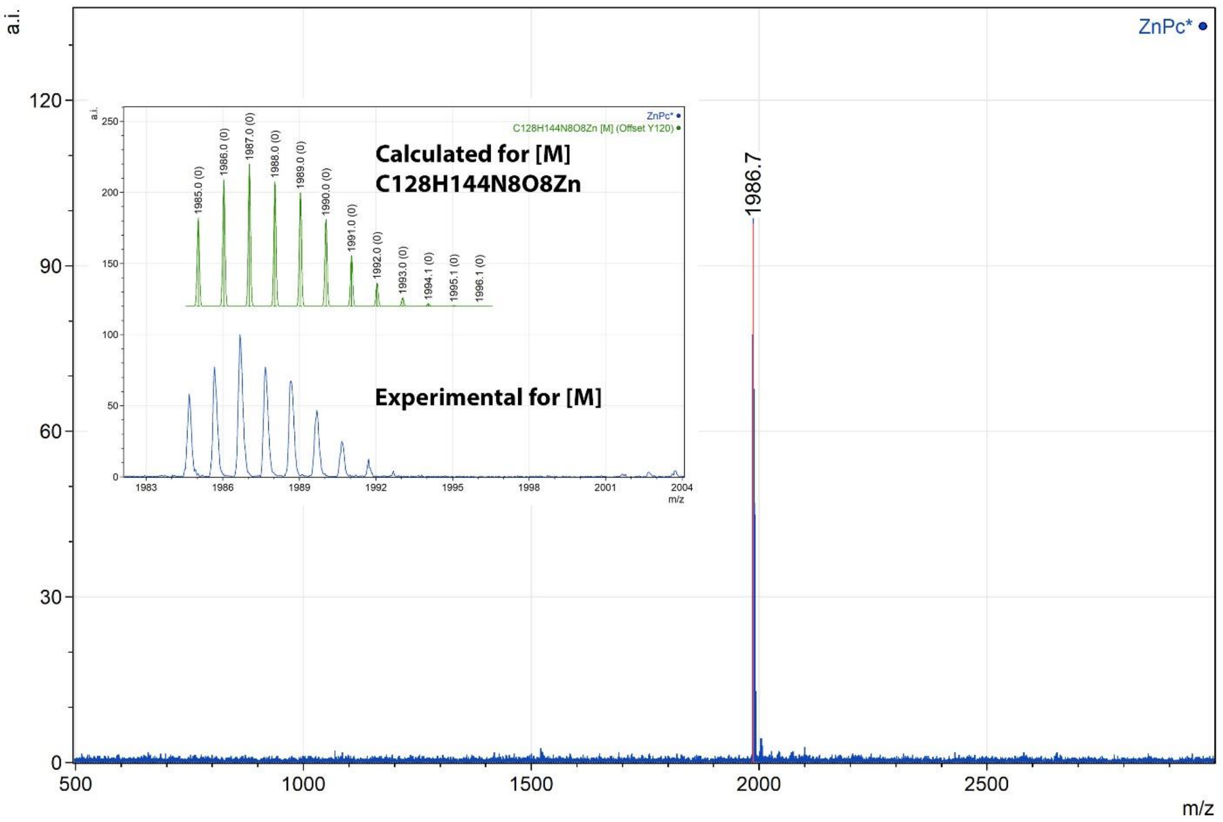

Figure S10. MALDI TOF mass-spectrum of **ZnPc***

Concentration dependence of UV-Vis

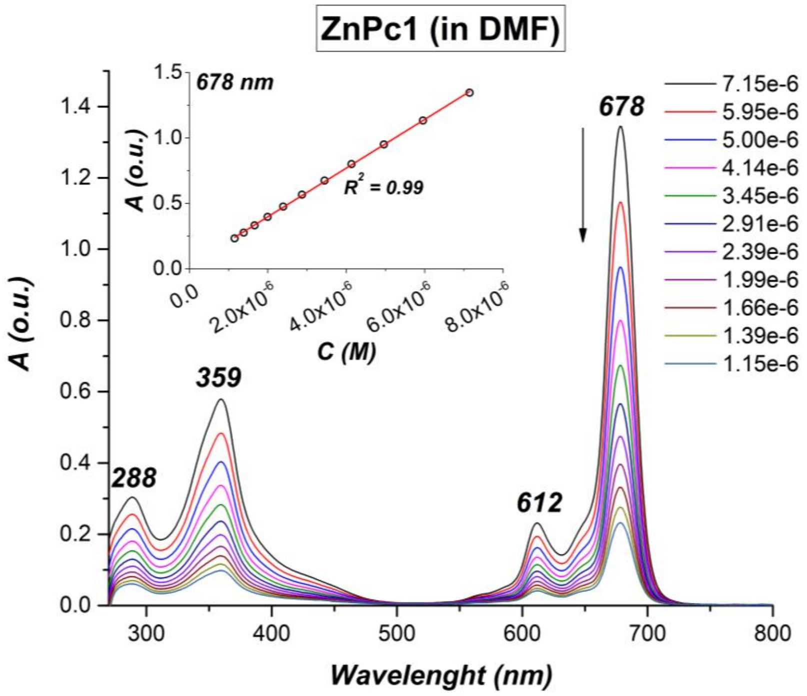

Figure S11. UV-Vis spectra at the various concentration of **ZnPc1** in DMF

The legend indicates concentration range. The arrow indicates decrease in concentration. The inset shows dependence of absorbance at 678 nm (Q-band) on concentration.

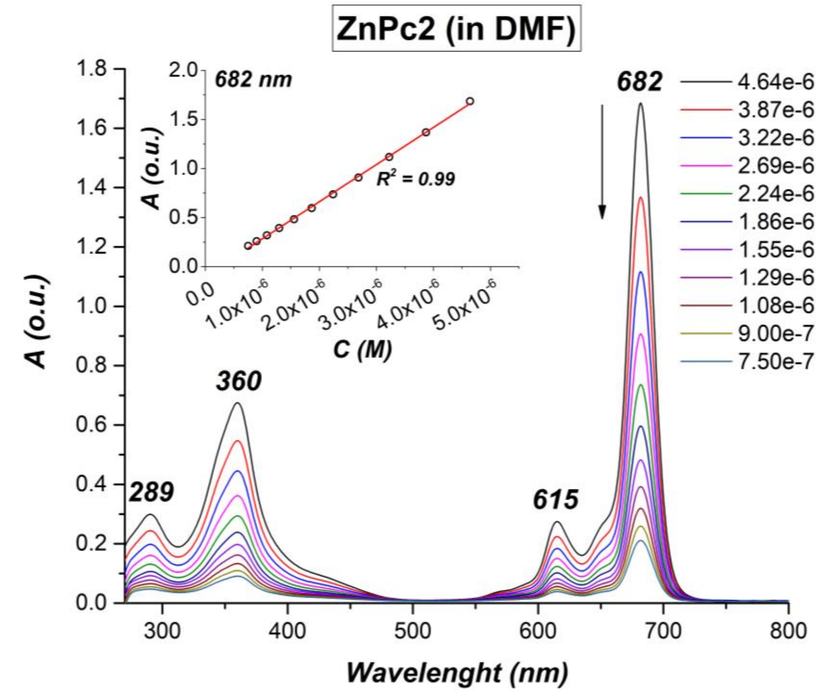

Figure S12. UV-Vis spectra at the various concentration of **ZnPc2** in DMF

The legend indicates concentration range. The arrow indicates decrease in concentration. The inset shows dependence of absorbance at 682 nm (Q-band) on concentration.

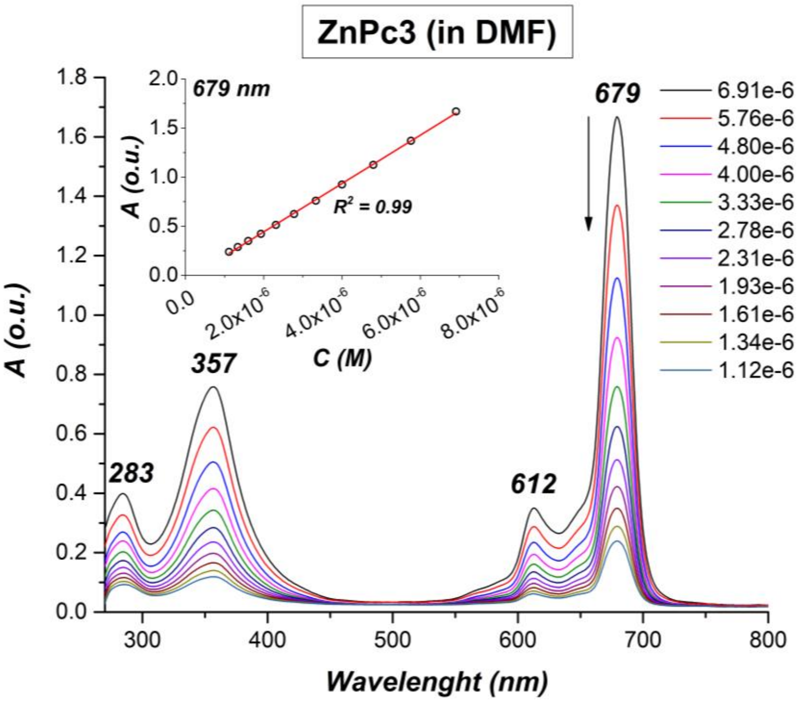

Figure S13. UV-Vis spectra at the various concentration of **ZnPc3** in DMF

The legend indicates concentration range. The arrow indicates decrease in concentration. The inset shows dependence of absorbance at 679 nm (Q-band) on concentration.

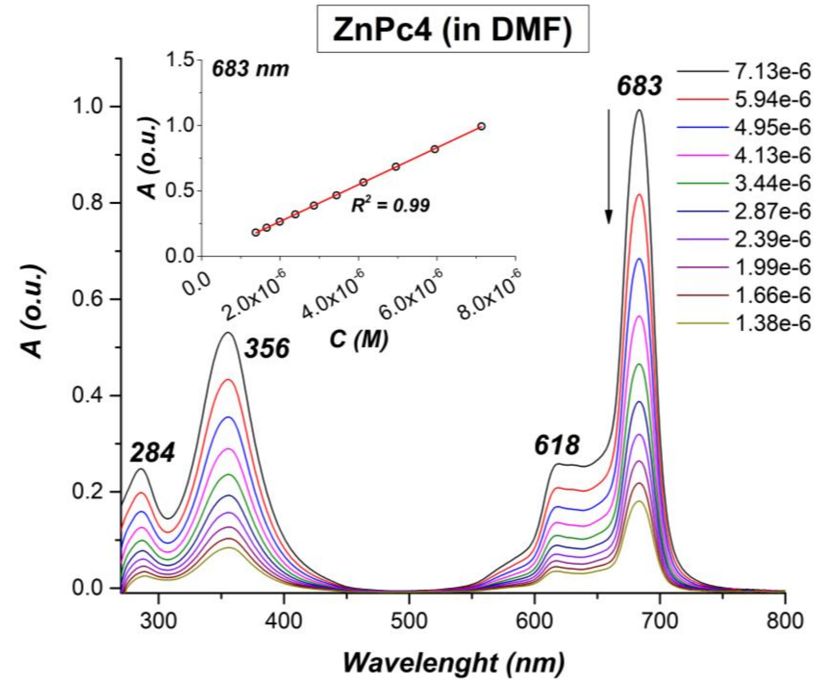

Figure S14. UV-Vis spectra at the various concentration of **ZnPc4** in DMF

The legend indicates concentration range. The arrow indicates decrease in concentration. The inset shows dependence of absorbance at 683 nm (Q-band) on concentration.

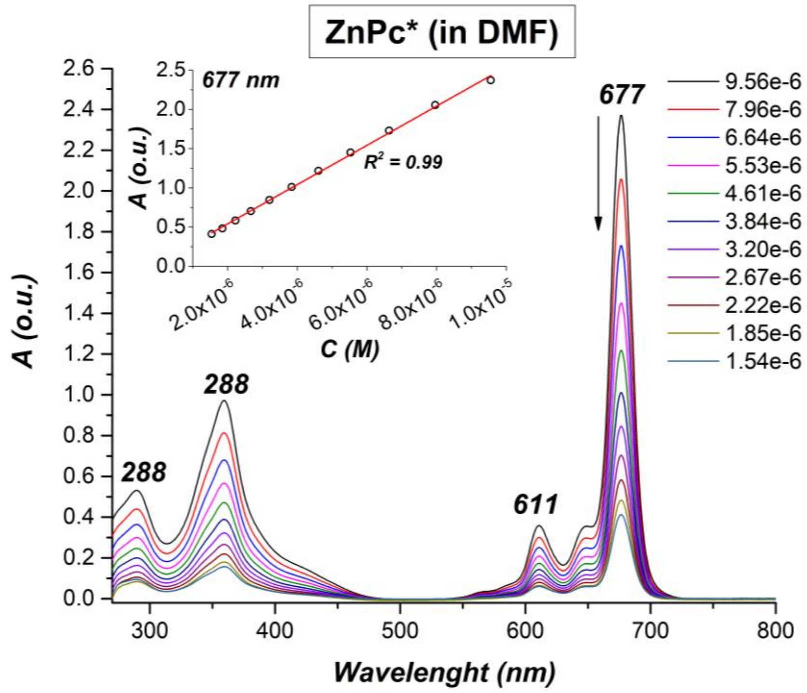

Figure S15. UV-Vis spectra at the various concentration of **ZnPc*** in DMF

The legend indicates concentration range. The arrow indicates decrease in concentration. The inset shows dependence of absorbance at 677 nm (Q-band) on concentration.

Cartesian coordinates of computed structures

Table S1. Gas-phase geometry of **ZnPc1** optimized at B3LYP/6-31G(d) level of theory

C	-1.190400	-5.434600	-0.014000
C	-1.241200	2.731900	-0.029600
C	-0.884900	4.146300	-0.023900
C	0.518800	4.207900	-0.022900
C	0.997500	2.830100	-0.028500
C	1.190300	5.434500	-0.015400
C	0.425300	6.595400	-0.009500
C	-1.001100	6.532900	-0.010900
C	-1.661400	5.309300	-0.017900
N	-0.087000	1.986500	-0.031700
N	0.086900	-1.986500	-0.028500
N	1.986500	0.086900	-0.032400
N	-1.986600	-0.087000	-0.033800
C	2.732000	1.241100	-0.026500
C	2.830200	-0.997600	-0.025400
C	4.146300	0.884800	-0.015000
C	4.207900	-0.518900	-0.014300
C	-2.732000	-1.241100	-0.025900
C	-2.830200	0.997500	-0.028000
C	-4.146400	-0.884900	-0.014600
C	-4.208000	0.518800	-0.016100
C	5.309300	1.661300	-0.003500
C	6.532800	1.001000	0.008300
C	6.595300	-0.425400	0.009700
C	5.434500	-1.190500	-0.001800
C	-5.309300	-1.661300	-0.002100
C	-5.434600	1.190400	-0.004800
C	-6.595400	0.425300	0.007900
C	-6.532900	-1.001100	0.008400
N	2.285100	2.494400	-0.026100
N	-2.494500	2.285100	-0.027800
N	-2.285100	-2.494500	-0.024200
N	2.494400	-2.285200	-0.024200
C	1.241100	-2.732000	-0.026200
C	-0.997600	-2.830200	-0.026100
C	-0.518900	-4.208000	-0.020400
C	0.884800	-4.146400	-0.020700
C	1.661300	-5.309400	-0.015200
C	1.001100	-6.533000	-0.009300
C	-0.425400	-6.595500	-0.008600
O	-0.940000	-7.866900	-0.000800
O	1.625600	-7.754200	-0.003600
O	-7.754000	-1.625600	0.018300
O	-7.866800	0.939900	0.023500
O	-1.625700	7.754100	-0.003600
O	0.939900	7.866900	-0.002200
C	5.042400	8.575500	0.012200
C	4.354200	8.443300	1.215700
C	2.978700	8.181800	1.239800
C	4.360600	8.456200	-1.196300
C	2.985200	8.195100	-1.230500
C	2.325300	8.051400	0.002200
C	2.242400	8.097900	-2.558300
C	2.228700	8.069900	2.562400
C	2.316500	9.424500	-3.338900
C	2.751300	6.921800	-3.413400
C	2.299800	9.387200	3.359000
C	2.731900	6.883300	3.406300
C	-5.775100	8.095400	-0.031300
C	-5.072800	8.040500	-1.232600
C	-3.679300	7.903000	-1.252600
C	-5.090400	8.023000	1.179300

C	-3.697300	7.884800	1.217600
C	-3.022100	7.815000	-0.013200
C	-2.954800	7.837000	2.548300
C	-2.917300	7.874700	-2.572800
C	-3.151700	9.141300	3.345200
C	-3.360200	6.608800	3.385200
C	-3.109300	9.186500	-3.358300
C	-3.304100	6.653700	-3.429000
C	-5.042600	-8.574800	-0.026800
C	-4.365800	-8.445600	1.183400
C	-2.990500	-8.184400	1.221100
C	-4.349400	-8.452800	-1.228500
C	-2.973700	-8.192200	-1.249200
C	-2.325400	-8.051300	-0.010000
C	-2.218300	-8.092100	-2.569700
C	-2.253000	-8.075800	2.551000
C	-2.290500	-9.414600	-3.357400
C	-2.714500	-6.909800	-3.423700
C	-2.327300	-9.396900	3.340900
C	-2.767700	-6.894600	3.395500
C	-8.575400	5.042100	-0.034600
C	-8.439900	4.375900	1.180800
C	-8.178600	3.000800	1.229200
C	-8.460300	4.338200	-1.230800
C	-8.199600	2.962400	-1.240800
C	-8.051600	2.325100	0.003200
C	-8.106000	2.195400	-2.554900
C	-9.430300	2.265500	-3.339800
C	-6.924100	2.680000	-3.416200
C	-8.094100	-5.774600	0.094100
C	-8.040400	-5.102300	-1.124400
C	-7.902900	-3.709800	-1.179100
C	-8.021100	-5.059900	1.287200
C	-7.883800	-3.666300	1.290800
C	-7.814700	-3.021900	0.043500
C	-7.836600	-2.890800	2.602500
C	-7.875300	-2.980800	-2.517800
C	-9.137300	-3.074700	3.408200
C	-6.603700	-3.268800	3.445400
C	-9.192600	-3.182900	-3.291600
C	-6.661500	-3.397200	-3.370500
C	5.775300	-8.094200	-0.011600
C	5.078800	-8.037500	-1.216300
C	3.685400	-7.900400	-1.242800
C	5.084600	-8.024100	1.195700
C	3.691400	-7.886600	1.227400
C	3.022100	-7.814800	-0.006500
C	2.942300	-7.841400	2.554500
C	2.929900	-7.870200	-2.566700
C	3.138800	-9.145200	3.352300
C	3.340400	-6.612300	3.393700
C	3.122500	-9.182800	-3.350700
C	3.323800	-6.650500	-3.421400
O	7.866700	-0.940000	0.023800
C	8.576300	-5.042400	0.017700
C	8.436500	-4.361500	1.224500
C	8.174200	-2.986300	1.255300
C	8.464000	-4.353300	-1.187400
C	8.202800	-2.977800	-1.214800
C	8.051300	-2.325400	0.020900
C	8.113200	-2.226900	-2.538600
C	8.054200	-2.244500	2.581800
C	9.442500	-2.300400	-3.314700
C	6.938800	-2.727400	-3.401000
C	9.369600	-2.313600	3.381800

C	6.867600	-2.758300	3.419300
O	7.754000	1.625500	0.019400
C	8.094600	5.775100	0.039300
C	8.042400	5.086300	-1.170000
C	7.905100	3.693200	-1.206100
C	8.019700	5.076700	1.241800
C	7.882000	3.683300	1.264200
C	7.814800	3.021900	0.025600
C	7.832200	2.925700	2.586200
C	7.879700	2.946100	-2.534800
C	9.133700	3.116300	3.389200
C	6.600700	3.319100	3.424100
C	9.196100	3.142000	-3.311800
C	6.664300	3.347000	-3.392500
C	-8.062900	2.275000	2.565100
C	-6.879800	2.799000	3.400800
C	-9.381800	2.352100	3.359000
H	-2.273200	-5.481000	-0.013200
H	2.273100	5.481000	-0.014200
H	-2.744000	5.260400	-0.018500
H	5.260400	2.743900	-0.003800
H	5.480900	-2.273200	-0.000800
H	-5.260400	-2.744000	-0.000600
H	-5.481000	2.273100	-0.005300
H	2.743900	-5.260500	-0.015500
H	6.109800	8.779500	0.016200
H	4.890800	8.549400	2.154800
H	4.902300	8.572400	-2.131300
H	1.187600	7.914400	-2.334500
H	1.174900	7.890100	2.330800
H	1.922400	10.255800	-2.744600
H	1.729300	9.357100	-4.262600
H	3.347900	9.670700	-3.618200
H	2.176000	6.849300	-4.344100
H	2.654100	5.970500	-2.879700
H	3.806300	7.049100	-3.683900
H	1.909600	10.225800	2.772400
H	3.329900	9.629100	3.646700
H	1.707600	9.309300	4.278600
H	2.151800	6.800700	4.333200
H	3.785700	7.006300	3.683600
H	2.636500	5.938500	2.861100
H	-6.856400	8.203100	-0.038400
H	-5.612800	8.109800	-2.173300
H	-5.644000	8.078800	2.112900
H	-1.886800	7.751800	2.328400
H	-1.852200	7.792900	-2.338100
H	-2.827600	10.012000	2.765200
H	-2.568400	9.113200	4.273400
H	-4.203000	9.292100	3.617900
H	-4.424000	6.637000	3.650400
H	-2.785800	6.576200	4.318700
H	-3.174900	5.677200	2.840300
H	-2.799900	10.052600	-2.763500
H	-4.156800	9.334400	-3.646600
H	-2.510800	9.172100	-4.277000
H	-4.364300	6.679600	-3.708400
H	-3.121400	5.717000	-2.891900
H	-2.716700	6.634600	-4.354800
H	-6.110000	-8.778400	-0.033500
H	-4.911300	-8.553800	2.117100
H	-4.882200	-8.566800	-2.168900
H	-1.164800	-7.914100	-2.335300
H	-1.197800	-7.891500	2.329900
H	-3.320100	-9.655100	-3.648100

H	-1.905800	-10.250500	-2.763400
H	-1.694100	-9.345100	-4.275000
H	-2.618300	-5.961500	-2.884500
H	-3.767600	-7.031600	-3.704300
H	-2.130500	-6.835200	-4.348800
H	-3.359400	-9.643600	3.617300
H	-1.744500	-9.321300	4.266700
H	-1.928600	-10.231500	2.754400
H	-2.670500	-5.947000	2.855400
H	-2.196200	-6.814300	4.327900
H	-3.823600	-7.022200	3.663000
H	-8.778900	6.109500	-0.049700
H	-8.542100	4.929600	2.110300
H	-8.579400	4.862700	-2.175200
H	-7.931200	1.143300	-2.311800
H	-9.668100	3.293400	-3.638500
H	-10.265900	1.888500	-2.740400
H	-9.365100	1.661100	-4.252500
H	-6.853700	2.087600	-4.336200
H	-5.974900	2.585500	-2.878300
H	-7.043100	3.731000	-3.705800
H	-8.201400	-6.855700	0.113900
H	-8.110200	-5.665500	-2.051300
H	-8.076100	-5.590300	2.234200
H	-7.758100	-1.828200	2.355800
H	-7.784500	-1.910800	-2.310100
H	-9.110200	-2.466600	4.320300
H	-9.280900	-4.119200	3.709500
H	-10.011700	-2.771600	2.822400
H	-6.571800	-2.672100	4.364800
H	-5.674700	-3.091300	2.893400
H	-6.625300	-4.326100	3.735800
H	-10.053200	-2.851100	-2.700800
H	-9.350000	-4.236500	-3.551400
H	-9.178400	-2.608900	-4.225900
H	-6.697000	-4.463100	-3.625700
H	-5.720900	-3.209800	-2.842100
H	-6.642200	-2.831200	-4.309500
H	6.856600	-8.201400	-0.013600
H	5.623400	-8.105200	-2.154400
H	5.633700	-8.081100	2.131800
H	1.875200	-7.759200	2.329400
H	1.863800	-7.785500	-2.337200
H	2.550500	-9.119000	4.277300
H	4.189000	-9.293100	3.630700
H	2.820300	-10.016800	2.770400
H	4.403100	-6.637700	3.663500
H	2.761800	-6.581600	4.324600
H	3.154900	-5.681000	2.848200
H	2.529200	-9.167100	-4.272800
H	2.807400	-10.047800	-2.757400
H	4.171200	-9.333600	-3.633100
H	4.385000	-6.679100	-3.696400
H	3.141300	-5.713200	-2.885300
H	2.740400	-6.630000	-4.349700
H	8.780700	-6.109700	0.016400
H	8.537300	-4.903800	2.160900
H	8.585900	-4.889300	-2.124900
H	7.932000	-1.172900	-2.309300
H	7.869600	-1.190400	2.355800
H	10.272800	-1.912700	-2.714900
H	9.380300	-1.707300	-4.234900
H	9.686500	-3.330800	-3.599600
H	6.871500	-2.146700	-4.328800
H	5.985800	-2.630500	-2.870300

H	7.064100	-3.781300	-3.677000
H	10.207700	-1.915200	2.800000
H	9.616200	-3.344400	3.663300
H	9.286000	-1.727900	4.305000
H	6.779300	-2.183700	4.349000
H	6.994800	-3.813000	3.691500
H	5.923900	-2.664600	2.871700
H	8.202000	6.856400	0.044600
H	8.113700	5.636900	-2.104400
H	8.073300	5.619800	2.181700
H	7.750100	1.860100	2.354000
H	7.792600	1.878700	-2.312500
H	10.006900	2.801700	2.807800
H	9.104400	2.521600	4.310000
H	9.280900	4.164600	3.675300
H	6.566900	2.734600	4.351300
H	5.671000	3.137500	2.874600
H	6.625800	4.380000	3.700600
H	10.058000	2.821700	-2.716700
H	9.349600	4.192400	-3.586400
H	9.183600	2.554900	-4.237800
H	6.647100	2.769300	-4.324300
H	6.695600	4.409800	-3.660800
H	5.724400	3.162500	-2.861600
H	-7.876500	1.218400	2.352200
H	-5.934400	2.701800	2.856600
H	-6.792700	2.233200	4.336100
H	-7.009000	3.856000	3.662900
H	-9.301800	1.777800	4.289700
H	-10.217200	1.945800	2.778800
H	-9.630800	3.385700	3.627100
Zn	-0.000000	-0.000100	-0.071800

Table S2. Gas-phase geometry of **ZnPc2** optimized at B3LYP/6-31G(d) level of theory

C	-5.379600	-0.124200	-0.013600
C	2.780400	0.177000	-0.017000
C	4.179900	0.590400	-0.008900
C	4.183200	1.994700	-0.003800
C	2.787700	2.416500	-0.011600
C	5.378400	2.717900	0.006300
C	6.572200	2.004200	0.009400
C	6.570300	0.575900	0.000500
C	5.374900	-0.136000	-0.007200
N	1.986800	1.295100	-0.019300
N	-1.987300	1.297900	-0.034700
N	0.000400	3.291500	-0.024300
N	-0.002200	-0.689900	-0.028600
C	1.124100	4.077000	-0.017400
C	-1.121600	4.081600	-0.024700
C	0.706900	5.470400	-0.011300
C	-0.702600	5.476400	-0.016800
C	-1.123600	-1.484200	-0.030900
C	1.117400	-1.487000	-0.022200
C	-0.706500	-2.884400	-0.026800
C	0.697300	-2.886200	-0.019200
C	1.412500	6.667500	0.008600
C	0.713400	7.885200	-0.006600
C	-0.721100	7.895700	-0.033400
C	-1.407700	6.676400	-0.012300
C	-1.433600	-4.077200	-0.028600
C	1.421000	-4.081000	-0.008900
C	0.705800	-5.275100	-0.008200
C	-0.721800	-5.273200	-0.021000
N	2.398000	3.684500	-0.009900
N	2.387200	-1.097400	-0.017900
N	-2.393000	-1.092800	-0.031900
N	-2.394700	3.688000	-0.028600
C	-2.785900	2.421700	-0.031500
C	-2.784200	0.182300	-0.031600
C	-4.182800	0.598700	-0.024600
C	-4.182800	2.003000	-0.026200
C	-5.374600	2.730800	-0.019300
C	-6.571200	2.020500	-0.008500
C	-6.572600	0.591800	-0.004100
O	-7.819000	0.024100	0.008000
O	-7.811900	2.595900	0.000800
O	-1.294000	-6.517000	-0.021500
O	1.274400	-6.520600	0.001000
O	7.815200	0.004700	0.002500
O	7.816000	2.577100	0.018000
C	1.565800	9.117600	-0.036100
O	1.335900	10.118300	-0.693100
O	2.704400	9.076500	0.686500
C	-1.657900	9.101900	-0.011800
O	-2.774100	8.984500	0.450000
O	-1.263000	10.255000	-0.552200
C	8.276400	6.715500	0.130100
C	8.208500	6.054900	-1.094200
C	8.034900	4.666600	-1.161300
C	8.184700	5.992300	1.317000
C	8.011300	4.602500	1.308900
C	7.926600	3.972000	0.055500
C	7.943000	3.816700	2.613600
C	7.993300	3.949000	-2.505900
C	9.257300	3.939700	3.409000
C	6.734200	4.234500	3.472300
C	9.333900	4.091100	-3.252800
C	6.818300	4.429200	-3.378600

C	8.332300	-4.124300	-0.132300
C	8.247600	-3.472800	1.095500
C	8.049900	-2.088300	1.169700
C	8.231600	-3.396500	-1.315500
C	8.035100	-2.010200	-1.300200
C	7.936400	-1.388100	-0.043600
C	7.958500	-1.218600	-2.600900
C	7.989700	-1.379000	2.517800
C	9.275900	-1.318500	-3.394500
C	6.757100	-1.649000	-3.463800
C	9.329500	-1.499400	3.269800
C	6.820200	-1.886000	3.382800
C	-8.349700	-4.102500	0.156500
C	-8.278200	-3.453300	-1.073400
C	-8.076500	-2.069700	-1.152500
C	-8.231300	-3.373100	1.337100
C	-8.029800	-1.987500	1.316900
C	-7.944600	-1.367900	0.058000
C	-7.934300	-1.193900	2.615000
C	-8.030200	-1.362800	-2.502400
C	-9.241100	-1.289300	3.426400
C	-6.722500	-1.625300	3.462800
C	-9.375900	-1.487600	-3.242800
C	-6.866800	-1.869000	-3.376200
C	5.401200	-7.046500	0.166400
C	4.757500	-6.971900	-1.066200
C	3.374000	-6.771700	-1.150800
C	4.666400	-6.932800	1.344100
C	3.280600	-6.733100	1.318400
C	2.666800	-6.644800	0.057000
C	2.481500	-6.642300	2.613500
C	2.673300	-6.721800	-2.503900
C	2.575100	-7.951400	3.421400
C	2.908300	-5.432600	3.466600
C	2.799700	-8.066500	-3.245900
C	3.185200	-5.557800	-3.373500
C	-5.423800	-7.027200	-0.163100
C	-4.774000	-6.945300	1.065800
C	-3.389200	-6.750300	1.142400
C	-4.694000	-6.926800	-1.345000
C	-3.307300	-6.732900	-1.327500
C	-2.687300	-6.636600	-0.069800
C	-2.513800	-6.656000	-2.627000
C	-2.682100	-6.692700	2.491800
C	-2.614300	-7.972600	-3.421900
C	-2.941600	-5.453300	-3.489400
C	-2.806600	-8.032900	3.242400
C	-3.188200	-5.522900	3.357000
C	-8.243400	6.739700	-0.025400
C	-8.158800	6.055900	1.184900
C	-7.996600	4.665400	1.223200
C	-8.182300	6.038100	-1.226800
C	-8.020900	4.647000	-1.247800
C	-7.917100	3.993500	-0.008300
C	-7.982700	3.885300	-2.568000
C	-7.934100	3.923300	2.553600
C	-9.313800	4.027400	-3.331700
C	-6.791500	4.315600	-3.444900
C	-9.258400	4.062500	3.329700
C	-6.738200	4.378500	3.411300
H	-5.381200	-1.208000	-0.011000
H	5.378400	3.801400	0.009800
H	5.372900	-1.219700	-0.012500
H	2.498500	6.643100	-0.012600
H	-2.490800	6.691900	0.014700

H	-2.517400	-4.074000	-0.035600
H	2.504700	-4.081100	-0.001500
H	-5.370100	3.814400	-0.020300
H	2.709700	8.282800	1.247500
H	-0.319600	10.225500	-0.848200
H	8.409900	7.793500	0.159200
H	8.295400	6.624400	-2.015600
H	8.254200	6.513300	2.268200
H	7.819200	2.760500	2.357700
H	7.845600	2.883400	-2.307700
H	10.113000	3.606300	2.812300
H	9.213800	3.325200	4.316100
H	9.446300	4.974700	3.717500
H	6.680800	3.621700	4.380000
H	5.794400	4.109400	2.923900
H	6.806000	5.283300	3.784400
H	10.165100	3.711600	-2.649000
H	9.547800	5.138100	-3.498100
H	9.309500	3.527200	-4.192900
H	6.783200	3.861600	-4.316000
H	6.915200	5.490200	-3.637800
H	5.860000	4.294900	-2.865600
H	8.486300	-5.199400	-0.167500
H	8.340200	-4.045600	2.014200
H	8.312200	-3.910800	-2.269400
H	7.819500	-0.165600	-2.339900
H	7.822300	-0.315500	2.324800
H	10.126000	-0.977000	-2.794400
H	9.225100	-0.698900	-4.297800
H	9.479500	-2.348800	-3.709600
H	6.844600	-2.694800	-3.782000
H	6.696100	-1.031400	-4.367800
H	5.814700	-1.542300	-2.916200
H	10.155800	-1.100800	2.671700
H	9.562300	-2.543600	3.510500
H	9.290500	-0.941200	4.212800
H	6.936900	-2.946300	3.637300
H	5.862000	-1.768800	2.865600
H	6.769700	-1.323600	4.322700
H	-8.507700	-5.176900	0.195500
H	-8.384500	-4.027400	-1.989900
H	-8.302200	-3.885300	2.292900
H	-7.796600	-0.141700	2.350400
H	-7.863700	-0.298500	-2.312800
H	-9.442800	-2.318600	3.746400
H	-10.098500	-0.947000	2.837200
H	-9.176600	-0.667900	4.327500
H	-5.787100	-1.520000	2.903200
H	-6.807400	-2.670700	3.782700
H	-6.649400	-1.006900	4.365300
H	-9.608400	-2.532600	-3.480400
H	-9.346300	-0.930300	-4.186600
H	-10.198100	-1.090200	-2.638200
H	-5.904600	-1.747500	-2.867500
H	-6.826000	-1.309100	-4.318000
H	-6.982900	-2.930400	-3.626300
H	6.475700	-7.202400	0.209700
H	5.335900	-7.074300	-1.980400
H	5.174600	-7.005500	2.301800
H	1.430200	-6.504900	2.345000
H	1.608400	-6.554100	-2.318900
H	3.603200	-8.152700	3.745200
H	2.236500	-8.807500	2.828200
H	1.950000	-7.890400	4.320300
H	2.286500	-5.362700	4.367000

H	2.804500	-4.495500	2.909400
H	3.952600	-5.517400	3.790200
H	3.845500	-8.299800	-3.479000
H	2.246800	-8.034700	-4.192300
H	2.398700	-8.889300	-2.644400
H	4.247500	-5.674800	-3.618900
H	3.062800	-4.596100	-2.864000
H	2.629800	-5.514700	-4.317900
H	-6.499200	-7.178300	-0.200000
H	-5.348500	-7.037400	1.983500
H	-5.207100	-7.004900	-2.299800
H	-1.461000	-6.518700	-2.364500
H	-1.617800	-6.527500	2.300800
H	-1.993600	-7.921900	-4.324400
H	-3.644600	-8.174600	-3.738400
H	-2.274800	-8.823800	-2.822100
H	-2.322800	-5.392500	-4.392600
H	-2.834500	-4.511500	-2.941000
H	-3.987000	-5.539200	-3.808900
H	-2.408900	-8.859600	2.644200
H	-3.851600	-8.263600	3.481200
H	-2.249800	-7.996000	4.186300
H	-4.249000	-5.637600	3.609500
H	-3.068300	-4.564500	2.840900
H	-2.627200	-5.474500	4.297800
H	-8.361200	7.819700	-0.032000
H	-8.219100	6.609700	2.117800
H	-8.261300	6.578000	-2.166500
H	-7.857200	2.824000	-2.334500
H	-7.799600	2.860000	2.334100
H	-9.292000	3.432400	-4.252600
H	-9.506200	5.069400	-3.613500
H	-10.157200	3.683600	-2.723400
H	-6.867100	5.369500	-3.737700
H	-6.758200	3.717600	-4.363500
H	-5.840600	4.181800	-2.918300
H	-9.219700	3.482800	4.259900
H	-10.104500	3.699400	2.736600
H	-9.459100	5.106800	3.596500
H	-6.821900	5.434900	3.692000
H	-5.791600	4.248700	2.876200
H	-6.688500	3.792400	4.336800
Zn	-0.001300	1.295400	-0.050100

Table S3. Gas-phase geometry of **ZnPc3** optimized at B3LYP/6-31G(d) level of theory

C	-5.460400	-2.155300	-0.014200
C	2.076400	0.961100	-0.075800
C	3.518800	1.165300	-0.039100
C	4.109600	-0.111900	-0.045500
C	3.012600	-1.075000	-0.087800
C	5.492300	-0.277900	-0.002500
C	6.290200	0.867700	0.050400
C	5.686300	2.154600	0.052400
C	4.307700	2.315100	0.008600
N	1.822600	-0.387500	-0.103900
N	-1.791900	-2.049700	-0.116500
N	0.846100	-3.028000	-0.122200
N	-0.815600	0.588300	-0.095300
C	2.194600	-3.278300	-0.110400
C	0.158400	-4.216400	-0.120700
C	2.400200	-4.722200	-0.106400
C	1.119800	-5.313600	-0.111100
C	-2.165600	0.842300	-0.082100
C	-0.128000	1.776900	-0.077100
C	-2.369100	2.286100	-0.056200
C	-1.091900	2.875800	-0.053100
C	3.554000	-5.502500	-0.093700
C	3.410200	-6.894600	-0.087700
C	2.122900	-7.480800	-0.071800
C	0.971700	-6.703000	-0.090900
C	-3.518800	3.075100	-0.030100
C	-0.924700	4.258200	-0.024200
C	-2.070900	5.057600	0.000400
C	-3.357800	4.454500	-0.002900
N	3.187100	-2.391900	-0.096000
N	1.188300	1.953000	-0.069100
N	-3.155700	-0.044500	-0.079500
N	-1.161100	-4.391000	-0.114200
C	-2.046900	-3.401800	-0.106600
C	-2.978700	-1.363200	-0.089900
C	-4.078300	-2.325100	-0.060000
C	-3.490400	-3.603200	-0.071300
C	-4.281700	-4.751300	-0.038500
C	-5.660100	-4.587900	0.008300
C	-6.261000	-3.299900	0.023800
O	-7.622400	-3.274600	0.079600
O	-2.046000	6.419800	0.034100
O	7.651900	0.843900	0.105300
C	4.585600	-7.826500	-0.055100
O	4.513700	-8.973300	0.318700
O	5.779400	-7.323100	-0.472300
Cl	6.713100	3.570200	0.116800
Cl	-4.773700	5.482300	0.029000
Cl	-6.689600	-6.001800	0.053700
C	9.771300	-2.741600	0.157100
C	9.330700	-2.177000	1.351800
C	8.600100	-0.981900	1.364700
C	9.498300	-2.110700	-1.054200
C	8.771700	-0.914000	-1.101500
C	8.326100	-0.386200	0.121900
C	8.502800	-0.215800	-2.430100
C	8.153000	-0.355900	2.681000
C	9.817200	0.184400	-3.127800
C	7.621800	-1.074700	-3.357600
C	9.363800	0.037800	3.549800
C	7.191500	-1.276500	3.456800
C	-9.742200	0.309700	0.165000
C	-9.305100	-0.268100	1.354600
C	-8.573700	-1.462700	1.356100

C	-9.464800	-0.306900	-1.052500
C	-8.737800	-1.502800	-1.111200
C	-8.296000	-2.044300	0.107500
C	-8.464300	-2.185600	-2.446800
C	-8.128800	-2.102800	2.666500
C	-9.776500	-2.579200	-3.152500
C	-7.581700	-1.315300	-3.361900
C	-9.340500	-2.506200	3.529400
C	-7.168500	-1.190200	3.453300
C	1.511500	8.573600	-0.106200
C	0.971500	8.198200	1.124000
C	-0.210200	7.452400	1.197500
C	0.873100	8.215500	-1.289100
C	-0.313400	7.468500	-1.275800
C	-0.818600	7.099100	-0.020700
C	-1.020400	7.103500	-2.576200
C	-0.859700	7.079200	2.526500
C	-1.475900	8.364300	-3.336600
C	-0.145600	6.202600	-3.468700
C	-1.968900	8.091100	2.884900
C	0.141100	6.936300	3.683500
H	-5.905100	-1.167400	-0.003300
H	5.940200	-1.264400	-0.003900
H	3.866600	3.305500	0.014700
H	4.524400	-5.014400	-0.049200
H	-0.015100	-7.154500	-0.085400
H	-4.509500	2.634600	-0.031000
H	0.062300	4.705400	-0.017800
H	-3.843200	-5.742900	-0.045900
H	5.650700	-6.452200	-0.881500
H	2.062900	-8.563400	-0.042800
H	10.337000	-3.669300	0.171300
H	9.562700	-2.667700	2.293100
H	9.859900	-2.549700	-1.980300
H	7.957900	0.707900	-2.216200
H	7.613400	0.566200	2.447300
H	10.428800	0.823900	-2.482600
H	9.605300	0.735300	-4.051700
H	10.415800	-0.694600	-3.394500
H	7.405600	-0.534100	-4.286600
H	6.667200	-1.324200	-2.881500
H	8.119400	-2.013800	-3.627500
H	10.030100	0.721600	3.013300
H	9.949900	-0.840400	3.845600
H	9.027700	0.537700	4.465800
H	6.848900	-0.782200	4.373500
H	7.679400	-2.214100	3.748300
H	6.309400	-1.528800	2.858500
H	-10.307700	1.237300	0.187700
H	-9.539900	0.211900	2.300800
H	-9.822000	0.143300	-1.974800
H	-7.919300	-3.111300	-2.241800
H	-7.588500	-3.022200	2.423700
H	-10.375300	-1.697800	-3.410500
H	-10.389100	-3.227000	-2.516500
H	-9.561400	-3.119100	-4.082200
H	-6.629600	-1.068300	-2.880000
H	-8.079800	-0.374100	-3.623500
H	-7.361400	-1.845800	-4.295700
H	-9.927200	-1.631400	3.834100
H	-9.005500	-3.016100	4.440300
H	-10.005900	-3.184200	2.984500
H	-6.285800	-0.931300	2.858700
H	-6.827200	-1.693800	4.365300
H	-7.657200	-0.255600	3.753400

H	2.430700	9.152200	-0.139200
H	1.478000	8.491300	2.037800
H	1.297200	8.522200	-2.241400
H	-1.922800	6.541100	-2.320100
H	-1.341800	6.103600	2.396900
H	-0.622100	8.977500	-3.648600
H	-2.127500	8.987300	-2.714800
H	-2.031800	8.084200	-4.239100
H	-0.694300	5.914600	-4.373100
H	0.148000	5.286800	-2.944400
H	0.769800	6.716700	-3.784900
H	-1.548000	9.094700	3.021100
H	-2.464500	7.801300	3.819100
H	-2.727100	8.141400	2.097900
H	0.584600	7.898700	3.964600
H	0.955400	6.247100	3.433600
H	-0.371700	6.547400	4.570100
Zn	0.014700	-1.217900	-0.178600

Table S4. Gas-phase geometry of **ZnPc4** optimized at B3LYP/6-31G(d) level of theory

C	5.409500	-2.161800	-0.006700
C	-1.934700	1.381800	0.063700
C	-3.362800	1.670200	0.036600
C	-4.027400	0.430100	0.039500
C	-2.988000	-0.596200	0.069400
C	-5.417200	0.344500	0.005500
C	-6.147600	1.535700	-0.034800
C	-5.468800	2.785000	-0.033700
C	-4.082900	2.864400	0.001200
N	-1.758500	0.021700	0.082500
N	1.752800	-1.846700	0.072900
N	-0.939000	-2.673600	0.083300
N	0.930800	0.843100	0.070800
C	-2.301300	-2.843700	0.077600
C	-0.318300	-3.896700	0.072900
C	-2.588200	-4.273000	0.066100
C	-1.343300	-4.932400	0.063300
C	2.293000	1.018700	0.055900
C	0.312900	2.069700	0.061600
C	2.579900	2.448800	0.037500
C	1.339400	3.111600	0.041800
C	-3.775700	-4.994600	0.027100
C	-3.745600	-6.395300	0.043100
C	-2.475500	-7.059400	0.068100
C	-1.284000	-6.320000	0.039300
C	3.773500	3.169100	0.012200
C	1.252900	4.501300	0.022400
C	2.444000	5.232800	-0.001400
C	3.693400	4.555800	-0.006700
N	-3.238500	-1.898800	0.072100
N	-0.990300	2.321700	0.059200
N	3.230100	0.076900	0.049100
N	0.989700	-4.147700	0.064500
C	1.930300	-3.212200	0.060500
C	2.976600	-1.229900	0.053700
C	4.019800	-2.252900	0.027200
C	3.360400	-3.495400	0.031600
C	4.085300	-4.686400	0.002400
C	5.471300	-4.601500	-0.031800
C	6.144600	-3.349800	-0.038600
O	7.505900	-3.400800	-0.079100
O	2.498800	6.594000	-0.027600
O	-7.507300	1.591500	-0.079300
C	-5.132100	-7.009600	-0.115700
O	-6.018400	-6.374900	-0.644200
O	-5.378300	-8.244800	0.344600
C	-2.221500	-8.532700	0.090900
O	-1.363600	-9.101500	-0.541100
O	-2.990900	-9.215800	1.001500
Cl	-6.410600	4.259100	-0.081600
Cl	5.166600	5.499000	-0.038900
Cl	6.418900	-6.071500	-0.070400
C	-9.814900	-1.877200	-0.105500
C	-9.363000	-1.333900	-1.305600
C	-8.573100	-0.177300	-1.327700
C	-9.494600	-1.262500	1.102300
C	-8.707000	-0.104800	1.140800
C	-8.251400	0.400300	-0.088200
C	-8.382600	0.575000	2.466400
C	-8.110500	0.425900	-2.649400
C	-9.663300	1.050900	3.179200
C	-7.542300	-0.334000	3.383700
C	-9.309500	0.863800	-3.513000
C	-7.190400	-0.535000	-3.426900

C	9.817100	0.064300	-0.115100
C	9.374400	-0.488900	-1.314300
C	8.581200	-1.643300	-1.332600
C	9.482400	-0.537200	1.095600
C	8.691600	-1.692600	1.137600
C	8.246700	-2.209300	-0.090900
C	8.354400	-2.360000	2.466300
C	8.128500	-2.258300	-2.652400
C	9.627900	-2.834100	3.193200
C	7.509800	-1.440500	3.369000
C	9.333300	-2.705200	-3.503200
C	7.214600	-1.304000	-3.444900
C	-0.922600	8.959600	0.142300
C	-0.419700	8.550300	-1.091300
C	0.711900	7.729900	-1.176200
C	-0.294500	8.562000	1.319300
C	0.843700	7.745100	1.294400
C	1.313100	7.342900	0.033800
C	1.542900	7.340800	2.587600
C	1.303400	7.326200	-2.522700
C	2.091600	8.572400	3.334200
C	0.623800	6.503600	3.497200
C	2.176300	8.465200	-3.088100
C	0.237200	6.887000	-3.540900
H	5.909800	-1.200800	-0.011800
H	-5.921500	-0.614400	0.004400
H	-3.584500	3.827300	-0.001600
H	-4.734200	-4.494100	-0.039600
H	-0.331700	-6.837200	0.008400
H	4.736400	2.670900	0.007200
H	0.293900	5.005400	0.022400
H	3.591000	-5.651400	0.003600
H	-4.601600	-8.625200	0.801300
H	-2.745600	-10.158000	0.919800
H	-10.422000	-2.778100	-0.112500
H	-9.629200	-1.813600	-2.243400
H	-9.862300	-1.686100	2.033000
H	-7.788300	1.466700	2.247300
H	-7.533900	1.327300	-2.422500
H	-10.242900	1.726800	2.541300
H	-9.409000	1.586800	4.101300
H	-10.310500	0.208800	3.451700
H	-7.281600	0.194000	4.308600
H	-6.611600	-0.642100	2.895300
H	-8.091200	-1.241400	3.661900
H	-9.947500	1.572800	-2.974600
H	-9.929600	0.008100	-3.805400
H	-8.959100	1.349600	-4.431300
H	-6.837700	-0.060000	-4.350000
H	-7.714900	-1.456200	-3.706500
H	-6.313700	-0.816700	-2.834100
H	10.431000	0.960800	-0.124800
H	9.653200	-0.021200	-2.254600
H	9.843600	-0.106800	2.025700
H	7.759000	-3.251500	2.250000
H	7.549400	-3.156800	-2.421200
H	10.275600	-1.992000	3.464600
H	10.210400	-3.517000	2.565700
H	9.364000	-3.361700	4.117300
H	6.584000	-1.133500	2.870700
H	8.059600	-0.532800	3.644900
H	7.239700	-1.960200	4.295900
H	9.956400	-1.853000	-3.799600
H	8.988800	-3.199600	-4.419000
H	9.966600	-3.409600	-2.953500

H	6.333600	-1.017400	-2.860600
H	6.867900	-1.786900	-4.366000
H	7.742100	-0.385800	-3.729500
H	-1.803300	9.594700	0.184800
H	-0.912400	8.875500	-2.002800
H	-0.689100	8.894600	2.275600
H	2.402600	6.719100	2.322400
H	1.962400	6.469100	-2.355100
H	1.285400	9.244800	3.650800
H	2.777200	9.145200	2.700900
H	2.637100	8.260600	4.232600
H	1.164500	6.187700	4.397100
H	0.266100	5.604800	2.983500
H	-0.253100	7.076700	3.820900
H	1.576500	9.364400	-3.273200
H	2.635900	8.163100	-4.036700
H	2.977100	8.730600	-2.390300
H	-0.416500	7.715400	-3.837400
H	-0.394200	6.086200	-3.140600
H	0.721100	6.514900	-4.451100
Zn	-0.003400	-0.912700	0.132400

Table S5. Gas-phase geometry of **ZnPc*** optimized at B3LYP/6-31G(d) level of theory

C	-1.190400	-5.434600	-0.014000
C	-1.241200	2.731900	-0.029600
C	-0.884900	4.146300	-0.023900
C	0.518800	4.207900	-0.022900
C	0.997500	2.830100	-0.028500
C	1.190300	5.434500	-0.015400
C	0.425300	6.595400	-0.009500
C	-1.001100	6.532900	-0.010900
C	-1.661400	5.309300	-0.017900
N	-0.087000	1.986500	-0.031700
N	0.086900	-1.986500	-0.028500
N	1.986500	0.086900	-0.032400
N	-1.986600	-0.087000	-0.033800
C	2.732000	1.241100	-0.026500
C	2.830200	-0.997600	-0.025400
C	4.146300	0.884800	-0.015000
C	4.207900	-0.518900	-0.014300
C	-2.732000	-1.241100	-0.025900
C	-2.830200	0.997500	-0.028000
C	-4.146400	-0.884900	-0.014600
C	-4.208000	0.518800	-0.016100
C	5.309300	1.661300	-0.003500
C	6.532800	1.001000	0.008300
C	6.595300	-0.425400	0.009700
C	5.434500	-1.190500	-0.001800
C	-5.309300	-1.661300	-0.002100
C	-5.434600	1.190400	-0.004800
C	-6.595400	0.425300	0.007900
C	-6.532900	-1.001100	0.008400
N	2.285100	2.494400	-0.026100
N	-2.494500	2.285100	-0.027800
N	-2.285100	-2.494500	-0.024200
N	2.494400	-2.285200	-0.024200
C	1.241100	-2.732000	-0.026200
C	-0.997600	-2.830200	-0.026100
C	-0.518900	-4.208000	-0.020400
C	0.884800	-4.146400	-0.020700
C	1.661300	-5.309400	-0.015200
C	1.001100	-6.533000	-0.009300
C	-0.425400	-6.595500	-0.008600
O	-0.940000	-7.866900	-0.000800
O	1.625600	-7.754200	-0.003600
O	-7.754000	-1.625600	0.018300
O	-7.866800	0.939900	0.023500
O	-1.625700	7.754100	-0.003600
O	0.939900	7.866900	-0.002200
C	5.042400	8.575500	0.012200
C	4.354200	8.443300	1.215700
C	2.978700	8.181800	1.239800
C	4.360600	8.456200	-1.196300
C	2.985200	8.195100	-1.230500
C	2.325300	8.051400	0.002200
C	2.242400	8.097900	-2.558300
C	2.228700	8.069900	2.562400
C	2.316500	9.424500	-3.338900
C	2.751300	6.921800	-3.413400
C	2.299800	9.387200	3.359000
C	2.731900	6.883300	3.406300
C	-5.775100	8.095400	-0.031300
C	-5.072800	8.040500	-1.232600
C	-3.679300	7.903000	-1.252600
C	-5.090400	8.023000	1.179300
C	-3.697300	7.884800	1.217600
C	-3.022100	7.815000	-0.013200

C	-2.954800	7.837000	2.548300
C	-2.917300	7.874700	-2.572800
C	-3.151700	9.141300	3.345200
C	-3.360200	6.608800	3.385200
C	-3.109300	9.186500	-3.358300
C	-3.304100	6.653700	-3.429000
C	-5.042600	-8.574800	-0.026800
C	-4.365800	-8.445600	1.183400
C	-2.990500	-8.184400	1.221100
C	-4.349400	-8.452800	-1.228500
C	-2.973700	-8.192200	-1.249200
C	-2.325400	-8.051300	-0.010000
C	-2.218300	-8.092100	-2.569700
C	-2.253000	-8.075800	2.551000
C	-2.290500	-9.414600	-3.357400
C	-2.714500	-6.909800	-3.423700
C	-2.327300	-9.396900	3.340900
C	-2.767700	-6.894600	3.395500
C	-8.575400	5.042100	-0.034600
C	-8.439900	4.375900	1.180800
C	-8.178600	3.000800	1.229200
C	-8.460300	4.338200	-1.230800
C	-8.199600	2.962400	-1.240800
C	-8.051600	2.325100	0.003200
C	-8.106000	2.195400	-2.554900
C	-9.430300	2.265500	-3.339800
C	-6.924100	2.680000	-3.416200
C	-8.094100	-5.774600	0.094100
C	-8.040400	-5.102300	-1.124400
C	-7.902900	-3.709800	-1.179100
C	-8.021100	-5.059900	1.287200
C	-7.883800	-3.666300	1.290800
C	-7.814700	-3.021900	0.043500
C	-7.836600	-2.890800	2.602500
C	-7.875300	-2.980800	-2.517800
C	-9.137300	-3.074700	3.408200
C	-6.603700	-3.268800	3.445400
C	-9.192600	-3.182900	-3.291600
C	-6.661500	-3.397200	-3.370500
C	5.775300	-8.094200	-0.011600
C	5.078800	-8.037500	-1.216300
C	3.685400	-7.900400	-1.242800
C	5.084600	-8.024100	1.195700
C	3.691400	-7.886600	1.227400
C	3.022100	-7.814800	-0.006500
C	2.942300	-7.841400	2.554500
C	2.929900	-7.870200	-2.566700
C	3.138800	-9.145200	3.352300
C	3.340400	-6.612300	3.393700
C	3.122500	-9.182800	-3.350700
C	3.323800	-6.650500	-3.421400
O	7.866700	-0.940000	0.023800
C	8.576300	-5.042400	0.017700
C	8.436500	-4.361500	1.224500
C	8.174200	-2.986300	1.255300
C	8.464000	-4.353300	-1.187400
C	8.202800	-2.977800	-1.214800
C	8.051300	-2.325400	0.020900
C	8.113200	-2.226900	-2.538600
C	8.054200	-2.244500	2.581800
C	9.442500	-2.300400	-3.314700
C	6.938800	-2.727400	-3.401000
C	9.369600	-2.313600	3.381800
C	6.867600	-2.758300	3.419300
O	7.754000	1.625500	0.019400

C	8.094600	5.775100	0.039300
C	8.042400	5.086300	-1.170000
C	7.905100	3.693200	-1.206100
C	8.019700	5.076700	1.241800
C	7.882000	3.683300	1.264200
C	7.814800	3.021900	0.025600
C	7.832200	2.925700	2.586200
C	7.879700	2.946100	-2.534800
C	9.133700	3.116300	3.389200
C	6.600700	3.319100	3.424100
C	9.196100	3.142000	-3.311800
C	6.664300	3.347000	-3.392500
C	-8.062900	2.275000	2.565100
C	-6.879800	2.799000	3.400800
C	-9.381800	2.352100	3.359000
H	-2.273200	-5.481000	-0.013200
H	2.273100	5.481000	-0.014200
H	-2.744000	5.260400	-0.018500
H	5.260400	2.743900	-0.003800
H	5.480900	-2.273200	-0.000800
H	-5.260400	-2.744000	-0.000600
H	-5.481000	2.273100	-0.005300
H	2.743900	-5.260500	-0.015500
H	6.109800	8.779500	0.016200
H	4.890800	8.549400	2.154800
H	4.902300	8.572400	-2.131300
H	1.187600	7.914400	-2.334500
H	1.174900	7.890100	2.330800
H	1.922400	10.255800	-2.744600
H	1.729300	9.357100	-4.262600
H	3.347900	9.670700	-3.618200
H	2.176000	6.849300	-4.344100
H	2.654100	5.970500	-2.879700
H	3.806300	7.049100	-3.683900
H	1.909600	10.225800	2.772400
H	3.329900	9.629100	3.646700
H	1.707600	9.309300	4.278600
H	2.151800	6.800700	4.333200
H	3.785700	7.006300	3.683600
H	2.636500	5.938500	2.861100
H	-6.856400	8.203100	-0.038400
H	-5.612800	8.109800	-2.173300
H	-5.644000	8.078800	2.112900
H	-1.886800	7.751800	2.328400
H	-1.852200	7.792900	-2.338100
H	-2.827600	10.012000	2.765200
H	-2.568400	9.113200	4.273400
H	-4.203000	9.292100	3.617900
H	-4.424000	6.637000	3.650400
H	-2.785800	6.576200	4.318700
H	-3.174900	5.677200	2.840300
H	-2.799900	10.052600	-2.763500
H	-4.156800	9.334400	-3.646600
H	-2.510800	9.172100	-4.277000
H	-4.364300	6.679600	-3.708400
H	-3.121400	5.717000	-2.891900
H	-2.716700	6.634600	-4.354800
H	-6.110000	-8.778400	-0.033500
H	-4.911300	-8.553800	2.117100
H	-4.882200	-8.566800	-2.168900
H	-1.164800	-7.914100	-2.335300
H	-1.197800	-7.891500	2.329900
H	-3.320100	-9.655100	-3.648100
H	-1.905800	-10.250500	-2.763400
H	-1.694100	-9.345100	-4.275000

H	-2.618300	-5.961500	-2.884500
H	-3.767600	-7.031600	-3.704300
H	-2.130500	-6.835200	-4.348800
H	-3.359400	-9.643600	3.617300
H	-1.744500	-9.321300	4.266700
H	-1.928600	-10.231500	2.754400
H	-2.670500	-5.947000	2.855400
H	-2.196200	-6.814300	4.327900
H	-3.823600	-7.022200	3.663000
H	-8.778900	6.109500	-0.049700
H	-8.542100	4.929600	2.110300
H	-8.579400	4.862700	-2.175200
H	-7.931200	1.143300	-2.311800
H	-9.668100	3.293400	-3.638500
H	-10.265900	1.888500	-2.740400
H	-9.365100	1.661100	-4.252500
H	-6.853700	2.087600	-4.336200
H	-5.974900	2.585500	-2.878300
H	-7.043100	3.731000	-3.705800
H	-8.201400	-6.855700	0.113900
H	-8.110200	-5.665500	-2.051300
H	-8.076100	-5.590300	2.234200
H	-7.758100	-1.828200	2.355800
H	-7.784500	-1.910800	-2.310100
H	-9.110200	-2.466600	4.320300
H	-9.280900	-4.119200	3.709500
H	-10.011700	-2.771600	2.822400
H	-6.571800	-2.672100	4.364800
H	-5.674700	-3.091300	2.893400
H	-6.625300	-4.326100	3.735800
H	-10.053200	-2.851100	-2.700800
H	-9.350000	-4.236500	-3.551400
H	-9.178400	-2.608900	-4.225900
H	-6.697000	-4.463100	-3.625700
H	-5.720900	-3.209800	-2.842100
H	-6.642200	-2.831200	-4.309500
H	6.856600	-8.201400	-0.013600
H	5.623400	-8.105200	-2.154400
H	5.633700	-8.081100	2.131800
H	1.875200	-7.759200	2.329400
H	1.863800	-7.785500	-2.337200
H	2.550500	-9.119000	4.277300
H	4.189000	-9.293100	3.630700
H	2.820300	-10.016800	2.770400
H	4.403100	-6.637700	3.663500
H	2.761800	-6.581600	4.324600
H	3.154900	-5.681000	2.848200
H	2.529200	-9.167100	-4.272800
H	2.807400	-10.047800	-2.757400
H	4.171200	-9.333600	-3.633100
H	4.385000	-6.679100	-3.696400
H	3.141300	-5.713200	-2.885300
H	2.740400	-6.630000	-4.349700
H	8.780700	-6.109700	0.016400
H	8.537300	-4.903800	2.160900
H	8.585900	-4.889300	-2.124900
H	7.932000	-1.172900	-2.309300
H	7.869600	-1.190400	2.355800
H	10.272800	-1.912700	-2.714900
H	9.380300	-1.707300	-4.234900
H	9.686500	-3.330800	-3.599600
H	6.871500	-2.146700	-4.328800
H	5.985800	-2.630500	-2.870300
H	7.064100	-3.781300	-3.677000
H	10.207700	-1.915200	2.800000

H	9.616200	-3.344400	3.663300
H	9.286000	-1.727900	4.305000
H	6.779300	-2.183700	4.349000
H	6.994800	-3.813000	3.691500
H	5.923900	-2.664600	2.871700
H	8.202000	6.856400	0.044600
H	8.113700	5.636900	-2.104400
H	8.073300	5.619800	2.181700
H	7.750100	1.860100	2.354000
H	7.792600	1.878700	-2.312500
H	10.006900	2.801700	2.807800
H	9.104400	2.521600	4.310000
H	9.280900	4.164600	3.675300
H	6.566900	2.734600	4.351300
H	5.671000	3.137500	2.874600
H	6.625800	4.380000	3.700600
H	10.058000	2.821700	-2.716700
H	9.349600	4.192400	-3.586400
H	9.183600	2.554900	-4.237800
H	6.647100	2.769300	-4.324300
H	6.695600	4.409800	-3.660800
H	5.724400	3.162500	-2.861600
H	-7.876500	1.218400	2.352200
H	-5.934400	2.701800	2.856600
H	-6.792700	2.233200	4.336100
H	-7.009000	3.856000	3.662900
H	-9.301800	1.777800	4.289700
H	-10.217200	1.945800	2.778800
H	-9.630800	3.385700	3.627100
Zn	-0.000000	-0.000100	-0.071800
